# Experience-Dependent, Layer-Specific Development of Divergent Thalamocortical Connectivity

**DOI:** 10.1093/cercor/bhu031

**Published:** 2014-03-07

**Authors:** Alex Crocker-Buque, Sarah M. Brown, Peter C. Kind, John T.R. Isaac, Michael I. Daw

**Affiliations:** 1 Centre for Integrative Physiology, University of Edinburgh, Edinburgh, UK; 2 Developmental Synaptic Plasticity Section, National Institute of Neurological Disorders and Stroke, National Institutes of Health, Bethesda, MD 20892, USA; 3 Current address: Lilly UK, Erl Wood Manor, Windlesham, UK

**Keywords:** barrel cortex, LTP, plasticity, somatosensory

## Abstract

The main input to primary sensory cortex is via thalamocortical (TC) axons that form the greatest number of synapses in layer 4, but also synapse onto neurons in layer 6. The development of the TC input to layer 4 has been widely studied, but less is known about the development of the layer 6 input. Here, we show that, in neonates, the input to layer 6 is as strong as that to layer 4. Throughout the first postnatal week, there is an experience-dependent strengthening specific to layer 4, which correlates with the ability of synapses in layer 4, but not in layer 6, to undergo long-term potentiation (LTP). This strengthening consists of an increase in axon branching and the divergence of connectivity in layer 4 without a change in the strength of individual connections. We propose that experience-driven LTP stabilizes transient TC synapses in layer 4 to increase strength and divergence specifically in layer 4 over layer 6.

## Introduction

In the barrel cortex, ascending sensory input from the whisker pad enters neocortex by 2 distinct routes, the lemniscal and paralemniscal pathways (Castro-Alamancos 2004; Brecht 2007). The lemniscal input is the driving input to barrel cortex and lemniscal thalamocortical (TC) afferents synapse in L6A, L5B, and L4 (Bureau et al. 2006; Brecht 2007). The TC-L4 input comprises the highest density of TC synapses in the barrel cortex; it is precisely topographically mapped and mediates the temporally and spatially precise receptive fields observed in L4 and L2/3. The TC-L6A and TC-L5B inputs are weak in comparison (Bureau et al. 2006; Brecht 2007), although capable of inducing cortical spiking (Constantinople and Bruno 2013). TC-L6A is of interest because of the massive L6-thalamus projection that has been proposed to play a role in sensory gain control (Thomson 2010; Olsen et al. 2012). The difference in input strength between L4 and deeper layers is believed to be important for neocortical circuit function; however, it is not known when such a contrast in TC strength between L5, L6, and L4 is developmentally established and what physiological mechanism(s) underlie this process. Although sensory deprivation selectively affects the TC input to L4 over L6 in the visual cortex (Wang et al. 2013), it is not known whether this is also true in somatosensory cortex and whether sensory experience is required to initially establish L4 as the most densely innervated layer.

The precise topographic mapping of the TC input to L4 is refined during a critical period in the first postnatal week. *In vivo* this critical period is manifest as experience-dependent plasticity of whisker-receptive fields (Fox 1992), and *in vitro* experiments show that this corresponds to a critical period for the induction of long-term potentiation (LTP) at TC synapses (Crair and Malenka 1995; An et al. 2012). Also during this period, the thalamic input to L4 increases through a dramatic increase in the branching of TC axons (Agmon et al. 1993; Catalano et al. 1996). These highly branched axons form the substrate for highly divergent TC connectivity with each axon contacting approximately 50% of the cells within the barrel (Bruno and Simons 2002; Bruno and Sakmann 2006). Both experimental (Inan et al. 2006; She et al. 2009) and theoretical (Adams and Cox 2002) studies suggest that these new branches are involved in a Hebbian LTP-like process, whereby connections between specific pairs of thalamic and cortical cells are strengthened by experience. LTP produces both an increase in excitatory postsynaptic potential (EPSP) amplitude and a speeding of the kinetics of TC excitatory postsynaptic currents (EPSCs) (Kidd and Isaac 1999) and EPSPs resulting in precisely-timed, short latency action potentials in L4 cells (Daw et al. 2006), which is necessary for accurate processing of sensory information. It is not known how and when the dominance of the TC-L4 input over TC-L6 is established, or whether the TC-L6 input is subject to experience-dependent plasticity.

Here, we present the first demonstration that experience can drive an increase in input strength via increased divergence independent of the strength of individual connections. In the barrel cortex, this experience-dependent increase in divergence is specific to the TC input to L4, whereas the input to L6 is unchanged by sensory deprivation, and thereby establishes L4 as a major thalamorecipient layer by the end of the first postnatal week.

## Materials and Methods

### Electrophysiology

Five hundred-micrometer thick TC slices were prepared from P3 to P9 (P0 is designated as the day of birth) CD1, mouse pups as described previously (Agmon and Connors 1991; Daw, Ashby, et al. 2007). Briefly, mice were decapitated, and the brain removed and placed in an ice-cold solution containing either 119 mM NaCl, 2.5 mM KCl, 1 mM NaH_2_PO_4_, 26.2 mM NaHCO_3_, 11 mM glucose, 4.5 mM MgSO_4_, and 0.5 mM CaCl_2_ or a partial sucrose solution containing 80 mM NaCl, 3.5 mM KCl, 1.25 mM NaH_2_PO_4_, 25 mM NaHCO_3_, 10 mM glucose, 90 mM sucrose, 4.5 mM MgSO_4_, and 0.5 mM CaCl_2._ The brain was then cut at 45–50° to the midline and glued to the stage of a vibrating microtome on the cut surface. After cutting, slices were stored at room temperature for at least 1 h in cutting solution before recording. Slices were transferred to a recording chamber and perfused with an extracellular solution as follows: 119 mM NaCl, 2.5 mM KCl, 1 mM NaH_2_PO_4_, 26.2 mM NaHCO_3_, 11 mM glucose, 1.3 mM MgSO_4_, 2.5 mM CaCl_2_, and 5 µM picrotoxin to block gamma-aminobutyric acid A (GABA_A_) receptors, thus isolating monosynaptic TC EPSP/Cs from the powerful GABA_A_ receptor-mediated feedforward inhibition in the barrel cortex (Gabernet et al. 2005; Daw, Ashby, et al. 2007), and saturated with 95% O_2_/5% CO_2_, pH 7.4, at 33–35°C. Patch-clamp recordings were made from neurons in layer IV using infrared illumination and differential interference contrast optics. Whole-cell recordings were made with patch electrodes (4–7 MΩ) filled with 130 mM K-methanesulfonate, 8.5 mM NaCl, 5 mM 2-[4-(2-hydroxyethyl)piperazin-1-yl]ethanesulfonic acid (HEPES), 0.5 mM ethylene glycol-bis(2-aminoethylether)-*N*,*N*,*N*',*N*'-tetraacetic acid, 0.5 mM Na-GTP, and 4 mM Mg-ATP, pH 7.3, 285 mOsm. For perforated patch recordings, electrodes were tip-filled with the whole-cell solution and back-filled with the same solution including 200 µg mL^−1^gramicidin. For voltage-clamp recordings in minimal stimulation and CP93129 experiments, electrodes were filled with a solution containing 135 mM Cs methanesulfonate, 8 mM NaCl, 4 mM MgATP, 0.3 mM Na-GTP, 10 mM HEPES, and 5 mM QX314. TC EPSCs and EPSPs were evoked at a frequency of 0.1 Hz by electrical stimulation of TC axons by a bipolar stimulating electrode placed in the ventrobasal thalamus. Recordings were made using a Multiclamp 700A or B (Molecular Devices). Before making patch-clamp recordings, we probed the barrel field for TC responses with field electrode. Care was taken to ensure that the barrel with the largest field EPSP was selected to ensure that any differences between layers were not due to different lateral spread of TC inputs. For nonminimal stimulation, intensity was set such that the peak amplitude of the field EPSP was around 0.1–0.2 mV as this consistently results in EPSCs to be evoked in the vast majority of recorded L4 cells with an almost invariant onset latency and rising phase without kinks, indicating monosynaptic EPSCs without contamination from recurrent excitation. For dual L4–L5B/L6 experiments, stimulation intensity was adjusted to produce monosynaptic EPSCs in both cells and we included data only from recordings in which TC EPSCs were observed in both cells. A subset of CP93129 experiments in L6 was performed using minimal stimulation (see below), but the results did not differ from those in nonminimal stimulation, so data were pooled.

The L4/L5 boundary was identified based on the abrupt change in cell size and density. Namely L5 cells are large and sparsely distributed pyramidal cells and L4 cells are much smaller cells and more densely packed. We targeted cells with small, round somas in order to record spiny stellate cells [except at P3, and sometimes P4, when the majority of cells in L4 still display a pyramidal morphology (Callaway and Borrell 2011)] and usually recorded from cells just above L5 to avoid confusion with L2/3 especially in younger animals. Similarly, we targeted pyramidal cells in upper L6 as this is the main target of TC axons; the border of L6 was identified by a dense layer of smaller cells below L5. For L5B, we targeted large pyramidal cells in the lower third of L5. For 2-layer experiments using potassium methyl sulfate-based solutions at the end of experiments, we tested the firing response to a series of 500-ms depolarizing steps. As previously characterized (e.g., Daw, Ashby, et al. 2007), GABAergic interneurons have firing patterns that are readily distinguishable from principal cells, which in all layers (De la Rossa et al. 2013; Hedrick and Waters 2013) typically display shallow, slow AHPs, frequency adaptation, spike threshold accommodation, and substantial spike-broadening throughout spike trains. The vast majority of recorded cells selected on the basis of morphology showed principal cell-like firing patterns. Those that displayed interneuron-like firing patterns could also be identified by low membrane capacitance and bursts of fast, polysynaptic EPSCs. Cells with these properties were excluded from all analysis. While we and others have previously recorded from fast-spiking interneurons with large, monosynaptic EPSCs, we did not encounter these cells when selecting cells with the morphological characteristics described above. As such the vast majority of cells included in the analysis are stellate/pyramidal cells.

For LTP experiments, a pairing protocol was delivered after determining first that perforation had stabilized by monitoring series resistance then that the EPSC amplitude had been stable for at least 5 min. The pairing protocol consisted of 50 stimuli at 0.2 Hz while holding the postsynaptic cell held at 0 mV. For paired-pulse ratio, experiments 2 stimuli were delivered at 100 Hz interleaved with single stimuli at 0.2 Hz. Paired-pulse ratio was calculated as the peak amplitude of EPSC2/EPSC1. The peak of EPSC2 was determined by subtracting a peak-scaled EPSC from interleaved single-stimulus trials (see Fig. 4*A*). This is important for EPSCs with slow rise times when decay of EPSC1 significantly affects the observed peak of EPSC2. For minimal stimulation experiments, stimulus intensity was turned down until no EPSC was seen then increased until the minimum intensity at which an EPSC was observed. About 12–36 trials were recorded, depending on failure rate, and amplitude calculated from the peak amplitude of the average EPSC from all trials excluding failures (average of 11 ± 1 traces). Small increases in stimulation intensity typically resulted in a decrease in failure rate without a change in EPSC amplitude (see Fig. 4*B*), demonstrating that failures represent failures of axon stimulation. For dual minimal stimulation experiments, the intensity was that at which an EPSC was first seen in either cell. The average failure rate in these experiments of 0.47 and each experiment consisted of an average of 21 trials. The same axon was deemed to contact both cells if >65% of successes and 65% of failures were coincident. This level of coincidence was chosen as, assuming that all failures are failures of axon transmission, the false-positive rate for an experiment in which separate axons were stimulated contacting each cell would be <0.05 [given 11/21 successes and 10/21 failures in cell 1; *P* (≥7/11 coincident successes and ≥6/10 coincident failures in cell 2) = 0.04]. Coincident successes = 0.97 ± 0.02 when axon was deemed to contact both cells, *n* = 20; coincident successes = 0.11 ± 0.03 when same axon was not deemed to contact both cells. Signals were filtered at 4 kHz, digitized at 10 kHz, and stored on computer using the Signal 2 or Signal 4 software (Cambridge Electronic Design). We did not correct for junction potential. Series resistance (5–25 MΩ for whole cell, 20–50 M for perforated patch) was analyzed in the voltage clamp throughout the experiments and displayed on-line. Cells were rejected if series resistance changed by >20% during data collection. For perforated patch recordings, any sudden step change was taken as indicative of break-in and the recording discarded.

### Kinetic Analysis

Excitatory postsynaptic currents were fit with a dual exponential decay using the Signal 4 software and the fast tau used to categorize L6 EPSCs as the size and presence of slow components was highly variable. L4 cells were deemed to contain slow component only if both fast and slow tau exceeded 15 ms. The 10–90% rise time and EPSP half-width were calculated using scripts written in Signal 4.

### Axon Tracing

Daily whisker trims were performed on the right facepad starting within 24 h of birth. At postnatal day 8, mice were anesthetized with sodium pentobarbital (euthanol 200 mg/kg, i.p.) prior to transcardial perfusion with phosphate-buffered saline (PBS) followed by approximately 10 mL of 4% paraformaldehyde in 0.1 M phosphate buffer. Brains were removed from the skull and post-fixed in 4% paraformaldehyde for a minimum of 24 h. Pseudocoronal sections that maintain the TCA tract were mounted at 55° as described by Lee et al. (2005), and 250 µm sections were then cut on a vibrotome.

Small crystals of 1,1′-dioctadecyl-3,3,3′,3′-tetramethylindocarbocyanine perchlorate (DiI; Life Technologies, Carlsbad, CA, USA) were placed into the ventral posteromedial nucleus of the thalamus and left free floating in PBS for 14 days. L4 and barrel boundaries were identified by examining morphology and density of cells counterstained with TOPRO3 (1 : 1000; T3605; Invitrogen, Fig. 7*A*) Sections were imaged on an LSM510 Axiovert confocal microscope (Carl Zeiss). Three-micrometer confocal stacks were taken through L4 of the primary somatosensory cortex. DiI-labeled axons that left the section in the *z*-plane were not analyzed. The period of DiI transport required to fully label axons in L4 resulted in dense labeling in L6 such that individual axons were difficult to trace. We therefore analyzed individual TC axon branches entering L4 rather than entire TC axon arbors. This also required that axons in L6 were imaged in a separate set of experiments with shorter transport time. Sections containing back-labeled cells were discarded and only axons originating in the white matter were traced. As the L5/L6 border was not always readily identifiable in these sections, axons were traced from the white matter to 50% of the distance between the white matter and the more easily identified L4/L5 border. A maximum of 4 axons (mean 2.4 ± 0.2 axons) from each hemisphere were traced to provide a mean measurement for a single hemisphere. Inferential statistics were used to compare means derived from individual hemispheres; the number of replicates for each condition (*n*) was the number of hemispheres not the number of axons traced. Axons were reconstructed and measured using Simple Neurite Tracer plugin (Longair et al. 2011) in ImageJ (US National Institute of Health, Bethesda, MD, USA). Microscopy was done in the IMPACT Imaging Facility at the University of Edinburgh. The experimenter was blind to which hemisphere had been deprived until after analysis.

### Statistical Analysis

Unless otherwise stated, all statistical tests are 2-tailed *T*-tests (Microsoft Excel). Paired *T*-tests were used for kinetic parameters in dual recordings, LTP, and CP93129 experiments; unpaired *T*-tests were used for comparison between ages in dual recordings and between layers in minimal stimulation experiments. Wilcoxon paired signed-rank test (www.stattools.net) was used to test between amplitudes in dual recordings due to non-normal distribution of amplitudes.

Probabilities for the connection of an axon to 2 L4 cells between ages and for the proportion of EPSCs containing only a slow component were calculated using the binomial distribution to produce a *z*-value as follows:

$$z = \displaystyle{{{\,p_2}-{\,p_1}} \over {\hbox{SE}}},$$

where SE = √[*p*(1 − *p*) (1/*n*_1_ + 1/*n*_2_)]; pooled *p* = (*s*_1_ + *s*_2_)/(*n*_1_ + *n*_2_); *n_x_* = number trials in the condition *x*; *s_x_* = number successes in the condition *x*; *p_x_* = probability success in condition *x* (*s_x_*/*n_x_*).

All values given are mean ± SEM. *n* = the number of experiments unless otherwise stated.

### Drugs

Picrotoxin and gramicidin were purchased from Sigma and CP93129 from Tocris.

## Results

### Characterization of TC Input onto L6 Cells

**Figure 1.**
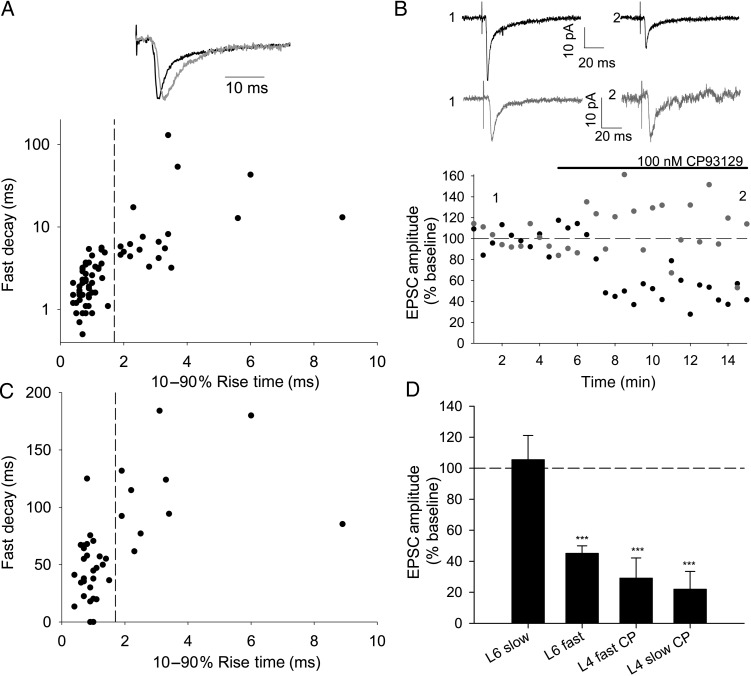
5-HT_1B_ agonist CP93129 inhibits thalamic-evoked L6 excitatory postsynaptic currents (EPSCs) with fast but not slow kinetics. (*A*) L6 thalamic-evoked EPSCs showing fast (black) and slow (gray) kinetics scaled to peak amplitude. Graph shows 10–90% rise time and fast decay (fast *τ* from a double exponential fit) for L6 EPSCs on a subset of which CP93129 was tested. Fast population (*τ*_fast_ 2.3 ± 0.2 ms, 10–90% rise time 0.8 ± 0.04, *n* = 51) and slow population (*τ*_fast_ 15.2 ± 6.4 ms, 10–90% rise time 3.1 ± 0.4, *n* = 21). Dashed line indicates rise time of 1.7 ms used to separate fast and slow populations. (*B*) Example experiments showing the effect of 100 nM CP93129 on fast (black) and slow (gray) EPSCs in L6. Traces show the average of 30 traces before (1) and after (2) application of CP93129 at time points shown. Points on this amplitude versus time plot and plot in *B* show peak amplitude of an average of 3 consecutive traces. Stimulus artifacts in this and following figures have been truncated. (*C*) Graph showing rise time versus EPSC amplitude as % baseline in CP93129 of all L6 cells tested. Dashed line indicates rise time of 1.7 ms used to separate fast and slow populations. (*D*) Summary graph showing the effect of CP93129 in conditions stated. ***P < 0.005.

Previously differences in conduction velocity and short-term plasticity (paired-pulse ratio) have been used to distinguish between TC EPSCs and EPSCs evoked by antidromic activation of axons of L6 cells, which project to ventral posteriomedial (VPM) thalamus in rats aged P14–P21 (Beierlein and Connors 2002). In neonatal (P3–P9) mice, however, we found that L6 EPSC latency, which is indicative of conduction velocity, decreased with age and that paired-pulse ratio was highly variable (data not shown). Thus, conduction velocity and paired-pulse ratio are not reliable indicators of TC inputs to L6 in the first postnatal week. In voltage-clamp recordings from L6 cells, we observed EPSCs with both fast (10–90% rise time <1.5 ms and decay *τ*_fast_ < 6 ms; Fig. 1*A*, black) and, less frequently, slow kinetics (rise time >1.8 ms, *τ*_fast_ > 3 ms; Fig. 1*A*, gray). 5-hydroxytryptamine 1B (5-HT_1B_) receptors are highly expressed in VPM cells (and therefore in terminals of TC axons in the cortex), but not in cells in the deep cortical layers in young animals (Bonnin et al. 2006). TC EPSCs in L4 are selectively inhibited by a 5-HT_1B_ agonist in the first postnatal week (Laurent et al. 2002), and presynaptic inhibition by these receptors has been used to distinguish EPSCs of thalamic and cortical origin in the neonatal thalamus (Evrard and Ropert 2009). Therefore, we used CP93129, a 5-HT_1B_ agonist, to define TC inputs to L6. We confirmed that 100 µM CP93129 strongly depresses TC EPSCs in L4 (fast EPSC in CP93129 29 ± 13% baseline, *n* = 5, *P* = 0.004; slow EPSC in CP93129 22 ± 12%, *n* = 5, *P* = 0.002, Fig. 1*D*) and also depresses fast EPSCs in L6 (45 ± 5% baseline, *n* = 29, *P* = 4 × 10^−12^; Fig. 1*B*,*D*), but has no effect on slow EPSCs in L6 (105 ± 16% baseline, *n* = 11, *P* = 0.7; Fig. 1*B*,*D*). The selective action of CP93129 on fast EPSCs in L6 cells shows that these fast currents (rise time <1.7 ms) are mediated by TC inputs, whereas slow EPSCs arise from a pharmacologically distinct population of synapses that is likely cortical in origin. This finding is in agreement with previous work, showing that EPSCs with fast kinetics are evoked in L6 by laser scanning photostimulation in VPM thalamus (Bureau et al. 2006). Similar slow EPSCs were observed in L5B, which were also insensitive to CP93129, whereas fast EPSCs in L5B were strongly depressed by 5-HT_1B_ activation (data not shown), so recordings in L5B cells were also restricted to those with fast EPSCs. Slow EPSCs in L4 have been characterized as being of TC origin (Kidd and Isaac 1999) and this is confirmed by their inhibition by CP93129.

### TC Input Strength Increases in Layer 4 Relative to Layer 6 During the First Postnatal Week

To determine whether L4 is already established as receiving the strongest TC input in neonatal somatosensory cortex, we made simultaneous whole-cell or perforated patch recordings from L4 and L5B or L6 cells while stimulating in VPM thalamus in slices prepared from mice aged P3–P5 (L2/3 is not fully formed at this age, so is not included in this study). Stimulation intensity was adjusted such that monosynaptic EPSCs were seen in both cells in all trials without contamination from recurrent inputs and slow, corticocortical EPSCs. In L5B, lower stimulation intensities often failed to produce EPSCs so higher intensities were required. In contrast, in L6, lower stimulation intensities were often required to avoid contamination from recurrent and/or slow EPSCs. Taken together, this resulted in larger L4 EPSCs in experiments involving L5B cells than those involving L6 cells. Importantly, variable stimulation intensities mean that only the relative input strength between layers in each experiment, rather than the absolute input strength, can be compared.

**Figure 2.**
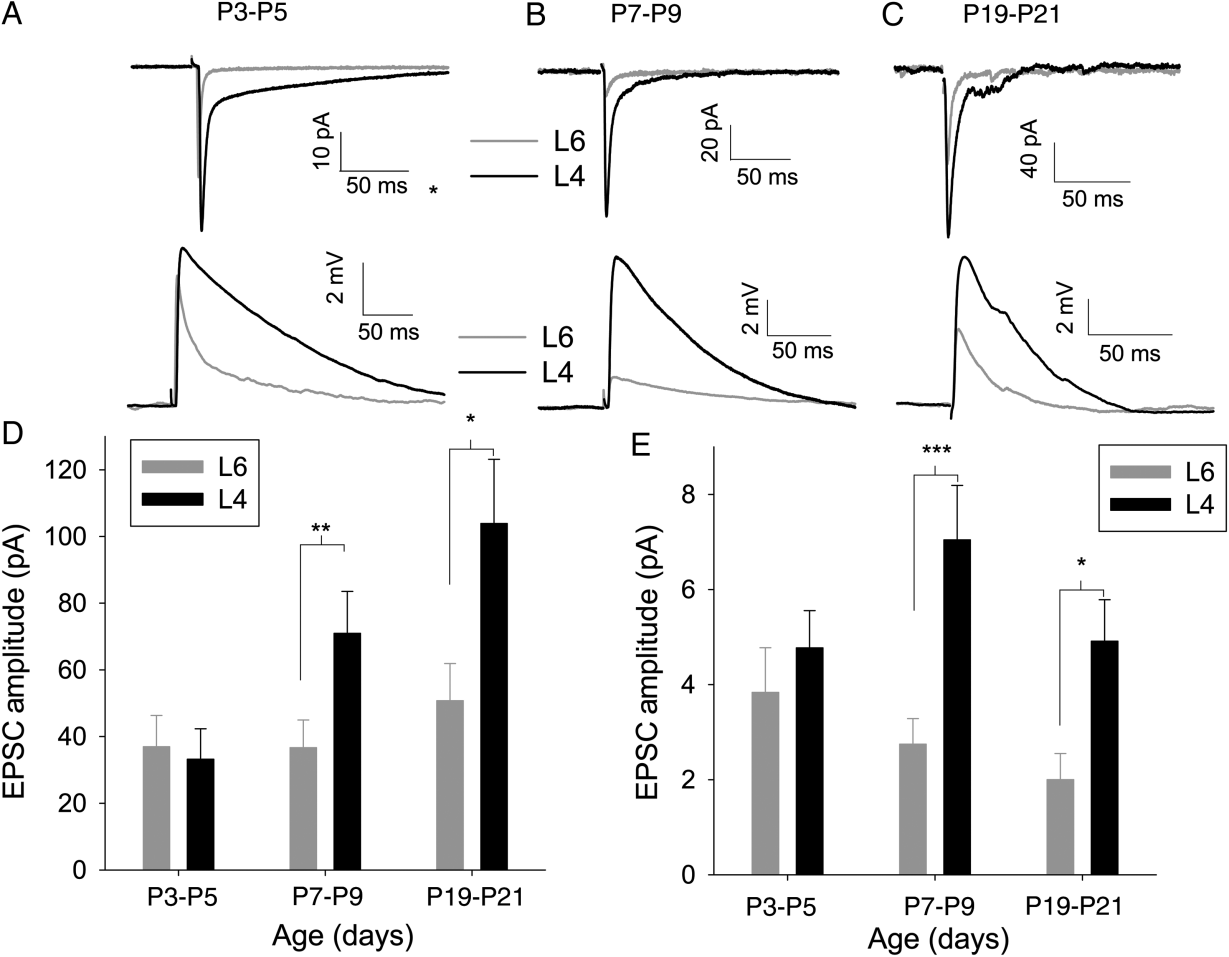
TC input to L4 increases relative to L6 during the first postnatal week. (*A*) Example traces showing an average of 15 TC EPSCs (upper) and excitatory postsynaptic potentials (EPSPs) (lower) in simultaneously recorded cells from mice aged P3–P5 in L4 (black) and L6 (gray). (*B*) As for *A* except mice aged P7–P9. (*C*) As for *A* except mice aged P19–P21. (*D*) Summary graph showing change in EPSC amplitude throughout the first week in L4 (black) and L6 (gray). (*E*) Summary graph showing change in EPSP amplitude throughout first week rise time throughout first week. *P < 0.5, **P < 0.01, ***P < 0.005.

At P3–P5, L6 receives an equally strong TC input to L4 with no difference in the amplitude of EPSCs (L4 33 ± 9 pA and L6 37 ± 9 pA, *n* = 17, *P* = 0.3; Fig. 2*A*,*D*) or EPSPs (L4 4.8 ± 0.8 mV and L6 3.8 ± 0.9 mv, *n* = 17, *P* = 0.2; Fig 2*A*,*E*). Although the presence of slow EPSCs in L4 would be expected to result in larger EPSPs (given equal peak EPSC) (Daw et al. 2006), this is balanced by much higher input resistance in L6 cells than in L4 (Table 1). To test if development during the first postnatal week leads to the larger input to L4, we repeated the simultaneous recordings in slices made from P7 to P9 mice. At this age, TC EPSCs in L4 cells were approximately double in amplitude compared with those in L6 cells (L4 74 ± 13 pA and L6 39 ± 9 pA, *n* = 11, *P* < 0.01; Fig. 2*B*,*D*). LTP at L4 TC synapses results in an increase in the fast EPSC, but a reduction in the slow EPSC, a change that is mirrored in development. These changes do not always result in an increase in peak EPSP amplitude (Daw et al. 2006); therefore, we investigated if the TC EPSP amplitude in L4 increases relative to L6. We found that EPSP amplitude in L4 is more than double that in L6 at P7–P9 (L4 7.2 ± 1.2 mV and L6 2.8 ± 0.6 mV, *n* = 11, *P* < 0.001; Fig. 2*B*,*E*). This apparent discrepancy is explained by a reduction in input resistance in L6 cells only (Table 1).

**Table 1:** Kinetic parameters of EPSPs and input resistance

		10–90% rise time (ms)			Half-width (ms)			Input resistance (MΩ)		
L4	P3–P5	**10**.**5**	*2*.*1*	*23*	**85**.**1**	*12*.*5*	*23*	**402**.**5**	38.4	25
	P7–P9	**3**.**1**	*0*.*3*	*22*	**44**.**2**	*4*.*2*	*22*	**385**.**5**	32.3	22
	P19–P21	**2**.**6**	*0*.*2*	*10*	**27**.**3**	*2*.*6*	*10*	**204**.**3**	15.0	10
L6	P3–P5	**5**.**1**	*1*.*9*	*13*	**88**.**2**	*20*.*2*	*13*	**871**.**1**	110.3	17
	P7–P9	**5**.**9**	*2*.*0*	*11*	**43**.**3**	*7*.*2*	*11*	**434**.**2**	65.8	11
	P19–P21	**1**.**2**	*0*.*1*	*10*	**18**.**3**	*1*.*6*	*10*	**199**.**2**	21.4	10

As per submission bold are actual values first italic column is +/− s.e.m. and 2nd italic column is n.

P3–P5 TC EPSPs in L4 had a slower rise time than in L6 indicative of the slow kinetic phenotype of juvenile TC EPSC/Ps in L4 (Daw et al. 2006). Speeding of kinetics in L4 meant that these differences were no longer present by P8–P9 (Table 1). This is consistent with L6-TC synapses having a mature kinetic phenotype at the earlier time point. Half-width was shorter at P8–P9 in both layers consistent with the loss of slow EPSCs in L4 and the decrease in input resistance in L6, which would result in a faster membrane time constant.

In contrast, we found that the input to L5B was much weaker than that to L4 at both P3–P5 (EPSC: L4 178 ± 53 pA, L5B 55 ± 10 pA, *P* < 0.01, Supplementary Fig. 1*A*,*C*; EPSP: L4 16.0 ± 5.0 mV, L5B 2.6 ± 0.7 mV, *n* = 10, *P* < 0.05, Supplementary Fig. 1*A*,*D*) and at P7–P9 (EPSC: L4 138 ± 338 pA, L5B 41 ± 10 pA, *P* < 0.01, Supplementary Fig. 1*B*,*C*; EPSP: L4 8.6 ± 2.1 mV, L5B 1.5 ± 0.4 mV, *n* = 10, *P* < 0.001, Supplementary Fig. 1*B*,*D*)

As the relationship between TC-L5B and TC-L6 lacks the clear developmental change shown between TC-L4 and TC-L6, we focused our subsequent investigations on the relative change in TC input to L4 and L6. The first postnatal week is critical for the development of TC-L4; however, it is possible that the strength of the TC input to L6 relative to L4 could change further after this age. To test this, we made further recordings from slices made from mice aged P19–P21. Similar to P7–P9, TC EPSCs and EPSPs in L4 cells were double the amplitude of those in L6 cells (EPSC: L4 103.9 ± 19.3 pA, L6 50.8 ± 11.1 pA, *n* = 10, *P* = 0.03, Fig. 2*C*,*D*; EPSP: L4 4.9 ± 0.9 mV, L6 2.0 ± 0.5 mV, *P* = 0.01, Fig. 2*C*,*E*), suggesting that the first postnatal week is the major period in which the dominance of the TC input to L4 is established. Input resistance decreased substantially between P7–P9 and P19–P21 in both layers (Table 1) resulting in a marked reduction in the EPSC : EPSP ratio.

### TC LTP Can Be Induced in L4 but Not in L6

**Figure 3.**
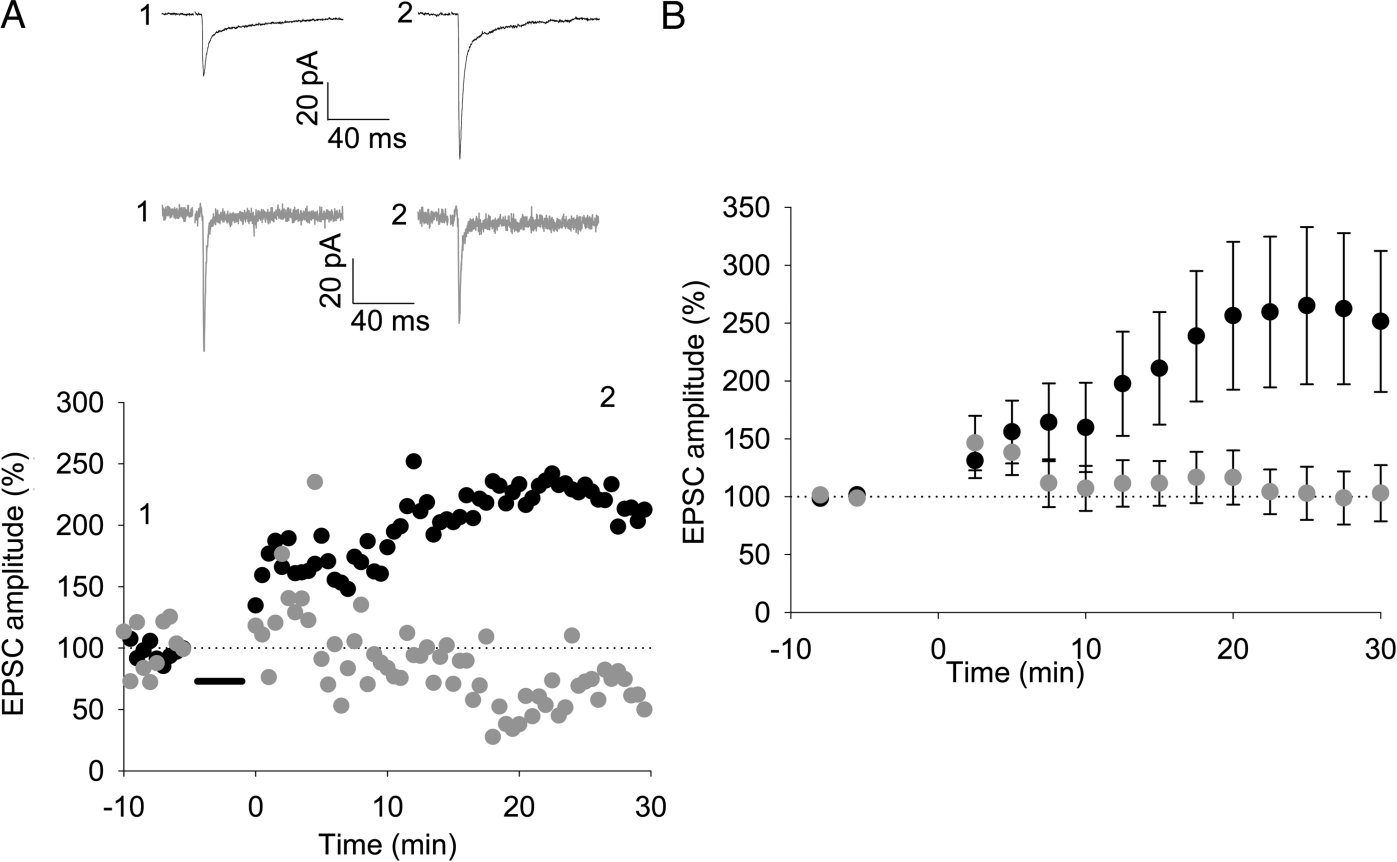
A pairing protocol induces LTP in L4 but not in L6 cells. (*A*) Upper panel: example traces from simultaneously recorded L4 (black) and L6 (gray) cells before (1) and after (2) pairing 50 stimuli at 0.2 Hz with a postsynaptic holding potential of 0 mV. Traces show an average of 3 sweeps. Lower panel: amplitude versus time plot for EPSCs from cells shown in *A*. Points in graph show peak amplitude from an average of 3 traces. (*B*) Summary graph showing the average of all experiments as shown in (*A*). Points show the average of 5 values as shown in (*A*).

Long-term potentiation is thought to underlie the developmental increase in L4 TC EPSC amplitude and speeding of EPSP rise time in L4 cells (Kidd and Isaac 1999; Daw et al. 2006). Given the increase in amplitude and speeding of kinetics of the TC input to L4 cells relative to L6 cells during the first postnatal week, we hypothesized that this form of LTP may not occur in L6 at this developmental stage. Previous studies in other brain areas have shown that LTP can be difficult to induce reliably with whole-cell recordings, but may be revealed by using perforated patch recordings (Lamsa et al. 2005). Therefore, we carried out an LTP pairing protocol (50 stimuli at 0.2 Hz with a postsynaptic holding potential of 0 mV) in simultaneous perforated patch recordings from L4 and L6 cells in slices from P3 to P5 mice. Robust LTP was induced in L4 cells (EPSC 25–30 min after pairing 257 ± 63%, *n* = 8, *P* = 0.041, Fig. 3*A*,*B*), whereas no LTP was observed in simultaneously recorded L6 cells (EPSC 101 ± 23% baseline, *n* = 8, *P* = 0.97, Fig. 3*A*,*B*). Additional single recordings made from L6 cells also showed no change in response to the pairing protocol (EPSC all L6 recordings 92 ± 14% baseline, *n* = 17, *P* = 0.6, Supplementary Fig. 2*A*). However, we found that the pairing protocol is sufficient to induce LTP of slow, putative corticocortical EPSCs in L6 cells (EPSC 203 ± 84% baseline, *n* = 7, *P* = 0.018, Supplementary Fig. 2*A*,*B*). Plotting change in EPSC amplitude in L6 versus EPSC rise time clearly shows that only slow, presumed corticocortical (CC), EPSCs are consistently potentiated. Taken together with the relative change in L4/L6 TC inputs, these data indicate that, although the TC input to L4 is plastic at P3–P5 and is developmentally regulated, L6 TC inputs do not readily express synaptic strengthening at an early postnatal age.

### No Developmental Change in Minimal Stimulation-Evoked EPSC Amplitude in L4 or L6

**Figure 4.**
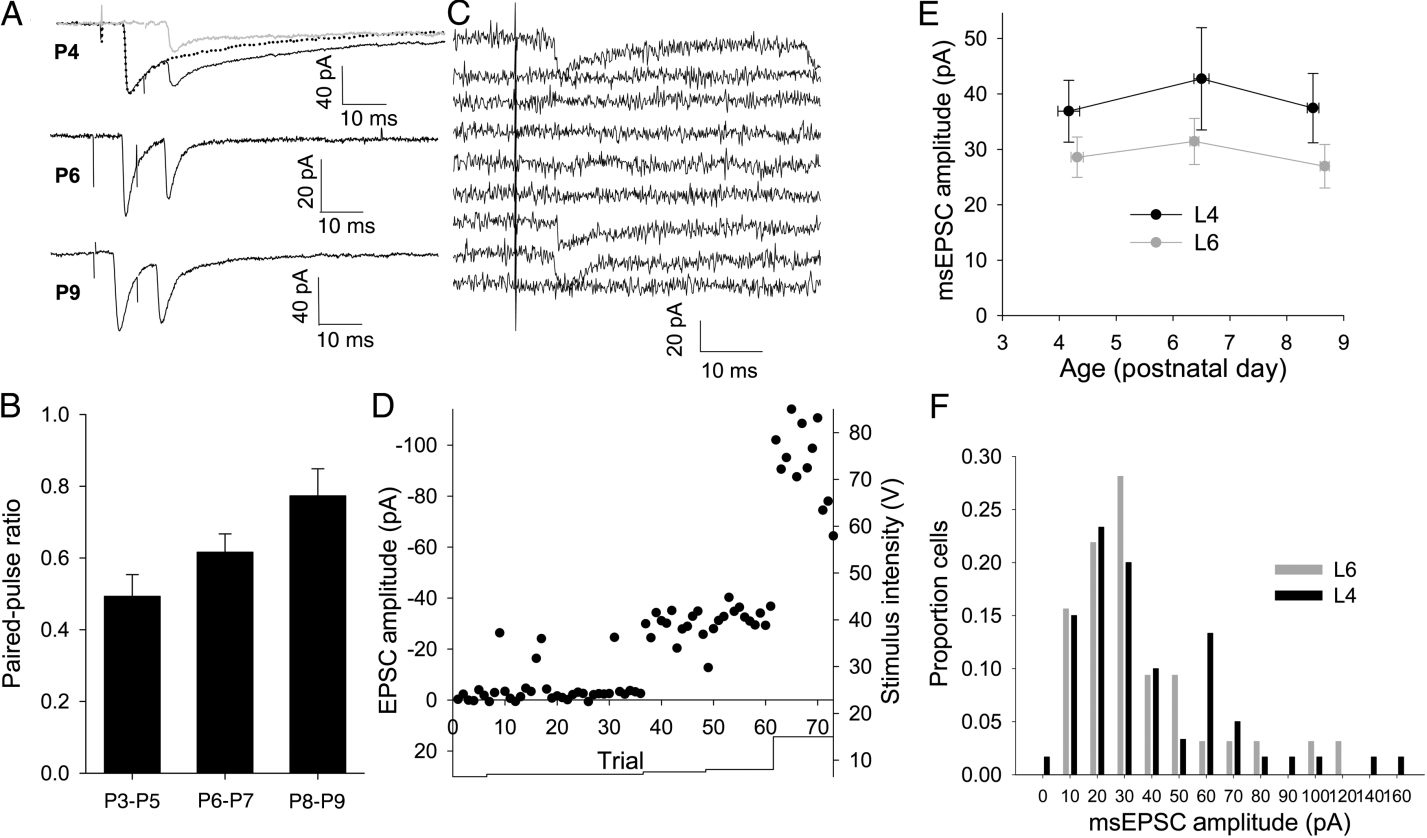
TC EPSCs evoked by minimal stimulation do not alter with age not true. (*A*) Example traces (average of 20 traces) showing paired stimuli at 100 Hz at different ages. Dotted line in top trace shows peak-scaled EPSC from interleaved trails. Gray line shows subtracted trace used to calculate peak of second EPSC. (*B*) Summary graph showing paired-pulse ratio at 100 Hz at P3–P5, P6–P7, and P8–P9. (*C*) Traces from 10 consecutive trials in an L4 cell with minimal TC stimulation intensity. In this case, 3 of the 10 trials evoked an EPSC. (*D*) Amplitude versus time plot for the experiment shown in (*C*). Points show peak amplitude for individual trials. Solid line shows stimulation intensity. (*E*) Summary graph showing EPSC amplitude from all minimal stimulation experiments in L4 (black) and L6 (gray) cells (L4 P3–P5 37 ± 6 pA, *n* = 18, P6–P7 43 ± 9 pA, *n* = 16, P8–P9 37 ± 6 pA, *n* = 26; L6 P3–P5 29 ± 4 pA, *n* = 35, P6–P7 31 ± 4 pA, *n* = 40, P8–P9 27 ± 4 pA, *n* = 36). (*F*) Amplitude distributions of msEPSC amplitude in all L4 (black) and L6 (gray) cells.

The above results show only that there is an increase in the TC-L4 relative to TC-L6 not whether there is an absolute increase in TC-L4 over the first postnatal week. An increase in TC-L4 could result from an increase in the quantal amplitude, an increase in the number of synapses between each connected TC-L4 cell pair, an increase in release probability, or an increase in the number of axons contacting a given cell. The quantal amplitude of L4 TC EPSCs remains constant throughout this period with a value of approximately 10 pA (Bannister et al. 2005). To investigate whether the probability of release increases with age, we recorded EPSCs in response to paired-pulse stimulation at 100 Hz in L4. We found that TC-L4 synapses show paired-pulse depression at all ages tested indicating a high probability of release (paired-pulse ratio P3–P5 = 0.49 ± 0.06, *n* = 8; P6–P7 = 0.62 ± 0.05, *n* = 6; P8–P9 = 0.77 ± 0.08, *n* = 8, *P* = 0.01, single factor ANOVA). The degree of depression, however, decreases with age suggesting a reduction rather than an increase in release probability consistent with a previous study (Kidd et al. 2002). Thus, any developmental increase in the strength of the TC input to L4 must be due to an increase in the number of axons contacting each cell or an increase in the number of functional release sites per TC axon onto each L4 cell. To test the latter possibility, we employed a minimal stimulation protocol during whole-cell recordings (Fig. 4*C*,*D*). Some TC EPSCs in L4 cells consist of only an immature slow kinetic EPSC (Kidd and Isaac 1999; Bannister et al. 2005); no change in the amplitude of these EPSCs was observed (data not shown, slow EPSCs excluded from further analysis). Minimal stimulation intensity was set by gradually increasing intensity from zero until the lowest intensity at which EPSCs were seen in a proportion of trials (Fig. 4*C*,*D*; mean failure rate was 0.42 ± 0.02) as previously described (Isaac et al. 1996, 1997). EPSCs evoked under these conditions minimal stimulation EPSC (msEPSC) are likely to be the result of stimulation of a single axon. We found no change in msEPSC amplitude in L4 (*P* = 0.8, Fig. 4*E*,*F*) or L6 (*P* = 0.6, Fig. 4*E*) throughout the developmental period studied. Importantly, although the amplitude of L6 msEPSCs is smaller than that of L4 msEPSC (*P* = 0.02), this difference is not large enough to account for the difference in EPSC amplitude seen during dual-cell recordings at P8–P9 (Fig. 2*H*) and does not vary with age (*P* = 0.42, Fig. 4*E*). This excludes the possibility that the increase in TC-L4 relative to TC-L6 is due to an increase in the number of functional release sites per TC axon.

### TC Axons Contact More L4 Cells After the First Week

**Figure 5.**
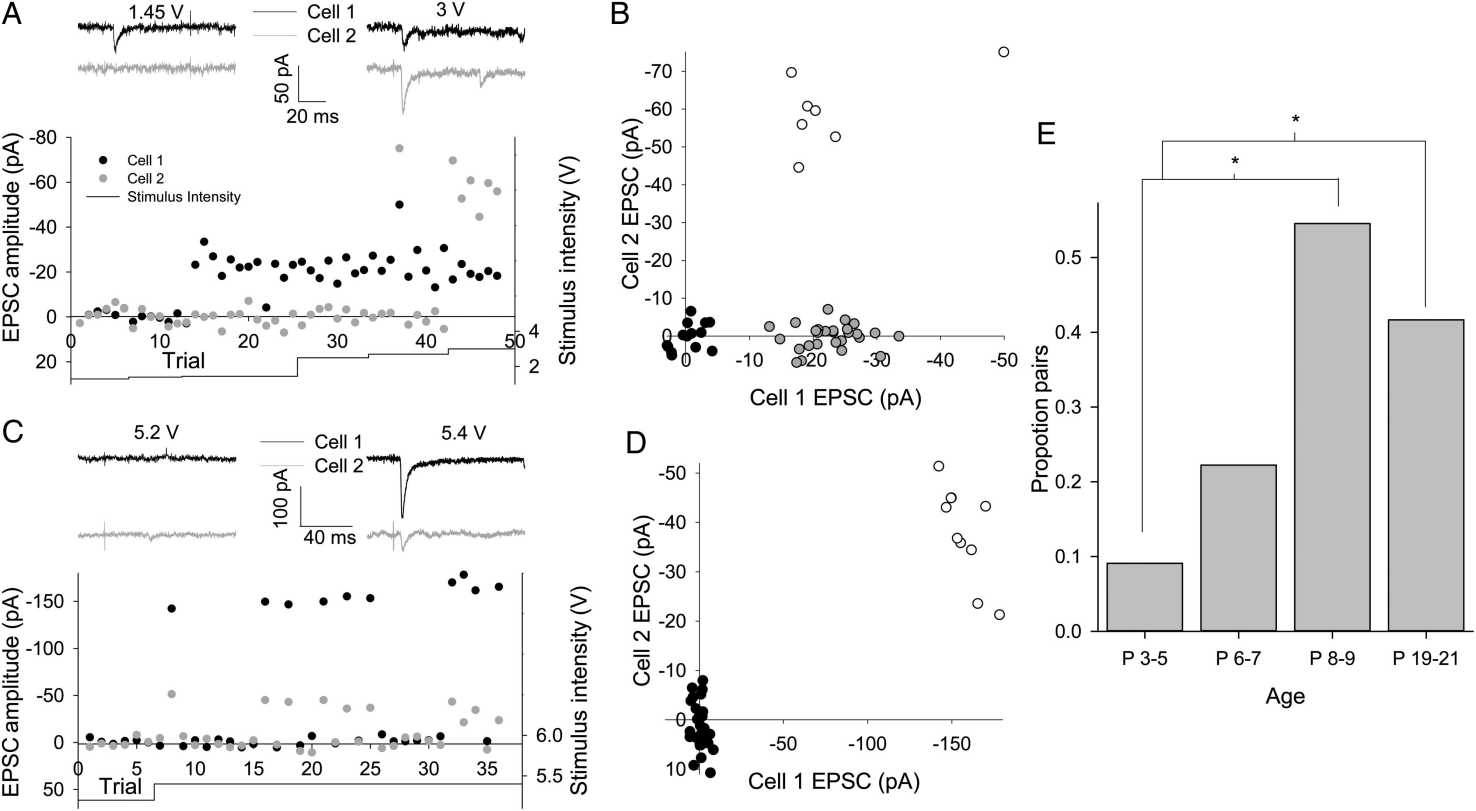
Increase in the proportion of L4 cells contacted by single TC axons with age. (*A*) Example experiment with 2 simultaneously recorded L4 cells during minimal TC stimulation. Traces show responses in cell 1 (black) and cell 2 (gray) to TC stimulation at intensity indicated. Lower panel shows amplitude and stimulus intensity versus trial no. plot for both cells. Note the failures in cell 2 on trials producing EPSCs in cell 1. (*B*) EPSC versus EPSC plot for all trials involving 2 cells shown in *A* (black circles no EPSC, gray circles EPSC in cell 1 only, and open circles EPSC in both cells). (*C*) As for *A* but 2 cells respond to same trial stimuli. (*D*) EPSC versus EPSC plot for all trials involving 2 cells shown in *C* (black circles no EPSC and open circles EPSC in both cells). (*E*) Bar graph showing the proportion of cell pairs responding to the same trials during minimal stimulation versus age. *P < 0.05.

We next tested the remaining possibility that selective strengthening of TC to L4 input is due to individual TC axons contacting a greater number of cells in L4. We took advantage of the high release probability to make the assumption that when a single axon is activated by minimal stimulation, an EPSC will be observed in all postsynaptic cells on almost every trial. We made simultaneous whole-cell recordings from 2 neighboring L4 cells and applied a minimal stimulation protocol to determine whether the same axon contacted each cell. Figure 5*A*,*B* shows an experiment in which the lowest stimulation intensity to evoke an EPSC in cell 1 failed to evoke an EPSC in cell 2. Successes in cell 1 coincide with failures in cell 2, which can be clearly seen when EPSC amplitude in cell 1 is plotted against that in cell 2 (gray circles in Fig. 5*B*). EPSCs are seen in cell 2 only with significantly higher stimulus intensity. In contrast, Figure 5*C*,*D* shows an experiment in which the same trials always produced either EPSCs or failures in both cells; here, the EPSC–EPSC plot shows that there are no trials that produce an EPSC in only one cell. Such recordings strongly suggest that the same axon makes synaptic contacts onto both recorded cells. The proportion of cell pairs contacted by the same axon increase sharply at the end of the first week with no further increase by P19–P21 (P3–P5 1/11, 9% pairs; P6–P7 2/9, 22% pairs; P8–P9 6/11, 55% pairs, *P* = 0.01 vs. P3–P5, P19–P21 5/12, 42% pairs, *P* = 0.04 vs. P3–P5, *P* = 0.27 vs. P8–P9; Fig. 5*E*); thus, an increase in the number of L4 cells functionally contacted by each TC axon in the absence of a change in the synaptic weight contributed by each axon is associated with the relative increase in TC input to L4 in the first postnatal week. To investigate TC connectivity to L6 cells, we recorded from 2 L6 cells simultaneously during minimal stimulation. Coincident EPSCs were never observed in 2 L6 cells (P3–P5 *n* = 13; P6–P7 *n* = 12; P8–P9 *n* = 14; P19–P21 *n* = 11) presumably because of low connectivity. As such it is not possible to conclude whether there is a change in the proportion of L6 cells contacted by each TC axon with development.

### Strengthening of TC-L4 Input Requires Whisker Experience

**Figure 6.**
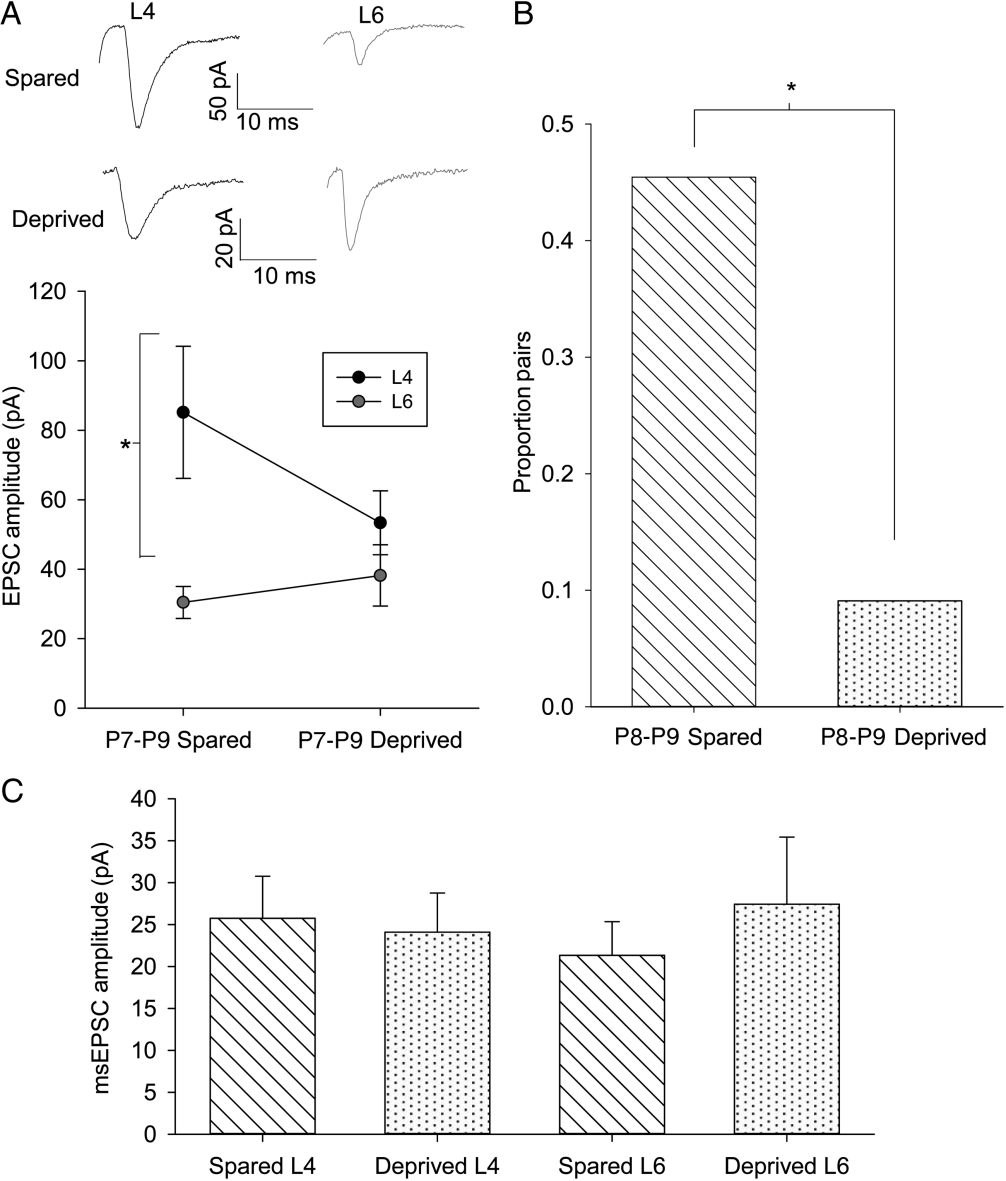
An increase in TC-L4 connectivity is experience-dependent. (*A*) Example traces and summary plot of simultaneously recorded cells in L4 (black) and L6 (gray) in sensory-deprived and -spared hemispheres from P7 to P9 mice. (*B*) Summary graph showing the proportion of L4 cells pairs responding to same TC axon during minimal stimulation. * *P* < 0.05. (*C*) Summary graph showing minimal stimulation EPSC amplitudes in L4 and L6 in spared and deprived hemispheres.

Experience-dependent plasticity plays a crucial role in tuning the specificity of somatosensory input to the cortex (Fox 1992). To determine whether the increase in relative strength of TC-L4 input is part of this experience-dependent plasticity, we produced sensory deprivation by trimming all of the whiskers on one side of the whisker pad on the face daily from P1 to the day of recording (P7–P9). TC slices were prepared from both hemispheres: contralateral (deprived) and ipsilateral (spared) to whisker trimming. First, we determined if whisker trimming prevented the relative increase in TC-L4 input. In recordings from the spared hemisphere of mice aged P7–P9, L4 cells exhibited a substantially larger TC EPSC than simultaneously recorded L6 cells (L4 85 ± 19 pA, L6 30 ± 5 pA, *n* = 10, *P* = 0.01, Fig. 6*A*) confirming our previous results (Fig. 2*B*,*D*). In contrast, in the deprived hemisphere, no difference was seen in the TC input strength between L4 and L6 (L4 53 ± 9 pA, L6 38 ± 9 pA, *n* = 11, *P* = 0.3, Fig. 6*A*). This finding suggests that whisker experience is required to drive the developmental increase in the number of L4 cells contacted by TC axons. We further tested this idea using minimal stimulation during simultaneous recordings from L4 cells. In the spared hemispheres, we found that a high proportion of P8–P9 L4 cell pairs are contacted by the same axon similar to our previous data set in control animals at the same age (5/11, 45% pairs; Fig. 6*B*). However, in deprived hemispheres, the proportion of L4 cell pairs contacted by the same TC axon was much lower and similar to that observed in controls at P3–P5 (1/11, 9% pairs, *P* = 0.03 vs. spared; Fig. 6*B*). Importantly, msEPSC amplitudes were not affected by whisker trimming in either layer (L4 EPSC_fast_ spared 25 ± 5 pA, *n* = 14, L4 deprived 25 ± 4 pA, *n* = 15, *P* = 0.9, L6 spared 21 ± 4, *n* = 7 and L6 deprived 27 ± 8, *n* = 6, *P* = 0.5; Fig. 6*C*). Therefore, these data show that whisker activity during the first postnatal week drives the selective strengthening of the TC input to L4 by promoting the divergence of the TC axons to L4 cells. As a result, the TC input to L6 remains as strong as that to L4 in the absence of whisker experience.

### Whisker Experience Promotes TC Axon Branching in Layer 4 but Not in Layer 6

**Figure 7.**
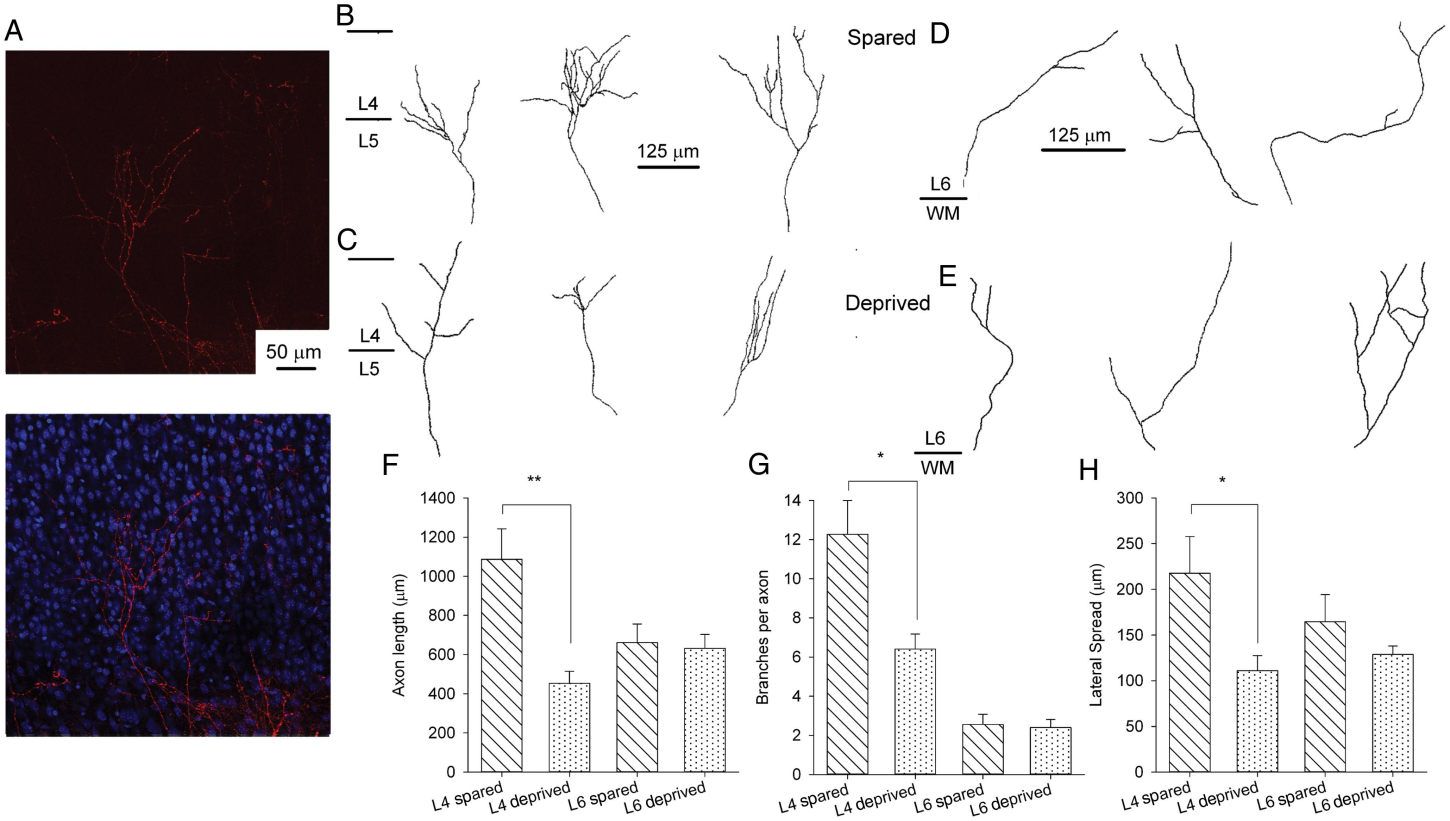
TC axon branching is reduced in deprived cortex. (*A*) Example images showing DiI labeling (red) of TC axons in cortex with overlaid Topro labeling (right image, blue) to identify cortical layers. (*B*) Example traced axons in L4 from the spared hemisphere. Middle tracing taken from image shown in *A*. (*C*) Example axon traces from L4 in the deprived hemisphere. (*D*) Example axon traces from L6 in the spared hemisphere. (*E*) Example axon traces from L6 in the deprived hemisphere. (*F*) Bar graph showing mean total TC axon length in L4 and L6 in both deprived and spared hemispheres. (*G*) Bar graph showing the mean lateral spread of TC axons in both deprived and spared hemispheres. (*H*) Bar graph showing the mean number of TC axon branches in L4 in deprived and spared hemispheres. * *P* < 0.05, **P < 0.005.

Our functional analysis strongly suggests that whisker experience drives a branching of TC axons in L4 producing an increase in divergence of this input onto L4 cells. To directly test this idea, we labeled TC axons by placing DiI crystals in the VPM of TC slices (Erzurumlu and Jhaveri 1992) cut from P8 to P9 mice after unilateral whisker trimming and compared TC axon collateral length and branching from deprived and nondeprived hemispheres. We found that both the total axon length and the number of branch points in L4 were strongly reduced in deprived hemispheres, compared with the spared hemispheres (total axon length in L4: spared hemisphere = 1086 ± 156 µm, *n* = 7, deprived = 453 ± 62 µm, *n* = 7, *P* = 0.002; Fig. 7*B*–*D*; branches per axon: spared = 12.3 ± 1.7, *n* = 7, deprived = 6.4 ± 0.8, *n* = 7, *P* = 0.01; Fig. 7*B*,*C*,*E*). In addition, the lateral spread of TC axon collaterals was also reduced in deprived hemispheres (spared = 218 ± 40 µm, *n* = 7; deprived = 111 ± 17 µm, *n* = 7, *P* = 0.03; Fig. 7*B*,*C*,*F*). Barrels vary considerably in size across the barrel field, but differences in axon parameters cannot be attributed to different barrel sizes (barrel width spared hemisphere = 155 ± 10 µm, *n* = 6, deprived 139 ± 10 µm, *n* = 5, *P* = 0.1). As we analyzed only single branches entering L4, we cannot rule out further differences in the number of these branches. If experience-driven strengthening of TC inputs is restricted to L4, we would expect whisker trimming to have no effect on TC axons in L6. Indeed, we found that there is no difference between spared and deprived hemispheres in terms of TC axon length, branching, or lateral spread in L6 (total axon length in L6: spared hemisphere = 661 ± 95 µm, *n* = 6, deprived = 632 ± 71 µm, *n* = 9, *P* = 0.8, Fig. 7*D*; branches per axon: spared = 2.6 ± 0.5, *n* = 6, deprived = 2.4 ± 0.4, *n* = 9, *P* = 0.8, Fig. 7*E*; lateral spread spared = 165 ± 30 µm, *n* = 6, deprived = 129 ± 9 µm, *n* = 9, *P* = 0.16, Fig. 7*F*). These findings confirm that whisker experience drives an increase in TC axon branching within individual barrels in L4, providing the anatomical substrate for the developmental increase in single axon functional divergence we observe.

## Discussion

The VPM thalamus receives sensory information from the whiskers and provides its major output via TC axons to L4 of the barrel cortex, but the same TC axons also make synapses onto cells in L5B and L6. VPM also receives direct feedback from L6 of the barrel cortex which is larger, in terms of axon and synapse number, than either its output to the cortex or its input from the lemniscal pathway (Castro-Alamancos 2004). Numerous studies have investigated mechanisms for the development (Daw, Scott, et al. 2007) and function (Brecht 2007) of the TC input to L4, but little is known about the changing synaptic phenotypes displayed by the TC input to L5B and L6 during development. Here, we show that the input to L5B is very weak from early in the first postnatal week and the properties of the TC input to L6 are fixed throughout the critical period of TC development, such that in neonates the thalamus drives L6 as strongly as L4 but by the end of the first postnatal week L4 receives a much larger input. EPSP rise time in L4 speeds up throughout this period, a process driven by LTP (Kidd and Isaac 1999; Daw et al. 2006). Accordingly, we find that a protocol that induces robust LTP in L4 cells does not affect EPSC amplitude in L6. Furthermore, the synaptic weight of individual TC axons to L6 cells is only slightly smaller than that to L4 cells, and this ratio does not change during the critical period. Instead, there is an experience-dependent increase in the number of TC axons contacting each cell within individual barrels producing the strong, divergent input to L4. A recent study has also demonstrated that early sensory deprivation, in the visual cortex, selectively induces plasticity at TC-L4 synapses without affecting L6 (Wang et al. 2013). Our study shows that this layer-specific, experience-dependent plasticity is a common feature of multiple sensory modalities achieved, in the somatosensory cortex, by increasing divergence and additionally demonstrates the relatively strong TC-L6 input early in development.

### Identification of TC Synapses

TC synapses have been distinguished from antidromically stimulated CC synapses based on axon conduction velocity and paired-pulse ratio (Beierlein and Connors 2002). We found that paired-pulse ratio was highly variable in these young animals, while EPSC latency showed a strong developmental reduction, presumably due to an increase in myelination, consistent with a previous study in L4 (Salami et al. 2003). We found, however, that a 5-HT_1B_ agonist consistently inhibited EPSCs with fast kinetics but not slow kinetics at all ages between P3 and P9. 5-HT_1B_ receptors are expressed at TC synapses at least until P10, but are absent beyond P14 and are not expressed in L6 cortical neurons at this age (Laurent et al. 2002). Thus, inhibition by 5-HT_1B_ agonists can be used as a reliable method for identification of TC synapses in neonates. Indeed, it has also been shown that 5-HT_1B_ receptor activation causes presynaptic inhibition at TC synapses on subplate neurons of neonates (Liao and Lee 2014).

### The Role of the TC Input to L6

It was recently found that L4 cells reprogrammed with an L5B-specific promoter had smaller TC EPSCs than neighboring L4 cells (De la Rossa et al. 2013). In agreement with this, we found that L5B cells had a much smaller TC EPSC than simultaneously recorded L4 cells at all ages tested. The input to L6, however, is as strong as that to L4 in neonates. We also find that the TC input to L6 does not display LTP from P3. As L6 develops before L4 it is possible that, in even younger animals, LTP could be induced in TC-L6 synapses. Furthermore, it is possible that TC synapses can be potentiated *in vivo*, but that we are unable to replicate the conditions which induce this potentiation. The lack of experience-dependent strengthening in L6 is supported, however, by the findings that in cortex deprived of sensory experience from P1 the TC input to L4 and L6 is still equal in strength by P7–P9 and TC axon length and complexity is different from cortex with normal sensory experience only in L4. Any change in L6 input strength must, therefore, occur in an experience-independent manner. It has been proposed that synchronized gamma oscillations in the thalamus and cortex are important for the development of the TC circuit by driving plasticity in L4 (Minlebaev et al. 2011). Feedback from layer 6 via the input to the inhibitory thalamic reticular nucleus and the VPM is likely to be necessary to maintain this synchronization.

### A Mechanism for the Activity-Dependent Increase in TC Input to L4

A standard Hebbian view of TC target refinement predicts that the strength of individual connections is increased by LTP (Adams and Cox 2002; Inan et al. 2006; She et al. 2009). Instead, we find that although experience drives an increase in TC axon branching and total strength of TC input to L4, the weight of individual TC-L4 connections does not change. Our data indicate that the increase in TC input to L4 cells relative to L6 is mediated by an increase in the number of axons contacting each cell. The distribution of amplitudes of msEPSCs, compared with the quantal amplitude at these synapses (Gil et al. 1999; Bannister et al. 2005), demonstrates that most axons make multiple synapses onto each cell contacted, with a stable number of contacts per cell throughout the age range studied.

**Figure 8.**
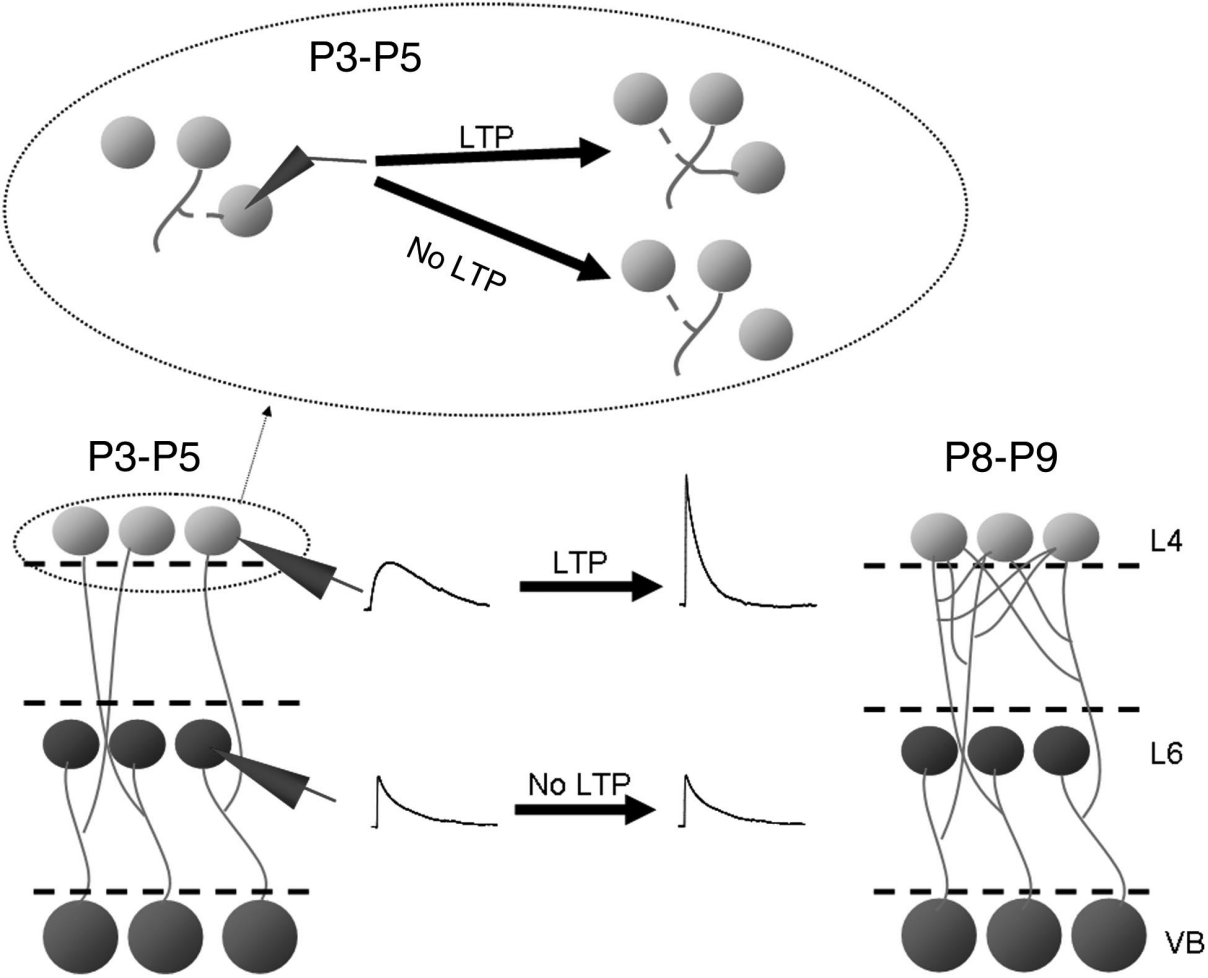
Development of TC Input to L4 and L6. Diagrammatic representation of findings showing the speeding of L4 TC EPSP kinetics and increase in amplitude resulting from LTP and an increase in the number of L4 cells contacted by each TC axon and the lack of relative change in L6 TC EPSP properties. The inset above shows that temporary synaptic contacts are made repeatedly in L4 during the P3–P5 period and only retained if that synapse undergoes LTP.

How can LTP mediate this increase in the number of TC axons contacting each cell? One explanation is that TC axons could make transient contacts preferentially onto cells which they do not already innervate, perhaps as immature synapses with slow kinetics (Kidd and Isaac 1999; Bannister et al. 2005). In this scheme, LTP would both convert these synapses to fast synapses and stabilize them against future elimination (Fig. 8). Further contacts requiring stabilization would then be made onto new cells. As such the decrease in the proportion of immature slow synapses (Kidd and Isaac 1999; Bannister et al. 2005) represents a developmental decrease in the number of new, transient contacts being formed, and the increase in the number of TC axons contacting each L4 cell is the result of multiple rounds of synapse stabilization by LTP. Whisker trimming did not result in an increased incidence of immature slow synapses at P8–P9 (data not shown), suggesting that the time period in which such transient contacts are formed is predetermined and this acts as the substrate for experience-dependent plasticity. Alternatively, or in parallel, temporary contacts may be mediated by NMDA-only silent synapses (Isaac et al. 1997). Silent synapses also decrease in number throughout the age range of this study, although we did not examine whether whisker trimming affected the developmental profile of silent synapse expression. Additionally, it is likely that we slightly underestimate the connectivity at P3–P5 as we did not test for silent synapses; however, this does not alter the finding that there was a large increase in the number of cells contacted by synapses functional at resting potential. Recently described potentiation of TC connections induced beyond the canonical critical period in response to adult sensory deprivation results in an increase in both mEPSC and msEPSC amplitude, suggesting that a separate mechanism is activated under these adult conditions (Yu et al. 2012). Our model is consistent with findings in the visual cortex in which light exposure in dark-reared mice initially results in an increase in spine turnover and motility (Tropea et al. 2010); equivalent to the turnover of transient synapses we propose in the barrel cortex. After 1 week of light exposure, spine turnover and motility decreases (Tropea et al. 2010) which could represent synapse stabilization by LTP. Furthermore, our data show that each TC axon is connected to approximately 50% of neighboring L4 cells. Anatomically individual TC axons branch extensively throughout the entire barrel without bias to one region (Jensen and Killackey 1987), suggesting that this highly divergent TC connectivity is retained throughout the entire barrel consistent with estimates of TC connectivity from *in vivo* recordings in adults (Bruno and Simons 2002). This similarity suggests that there is unlikely to be further experience-dependent increase in TC-L4 connectivity in animals older than those studied here. We cannot rule out, however, that there are later increases in the strength of TC input to L5B or L6. Such an increase may be implied from the demonstration in adult rats that L5B and L6 cells are strongly and directly activated by the thalamus *in vivo* (Constantinople and Bruno 2013). This study, however, also reports smaller sensory PSPs to deep layers than to L4 with a high level of sensory-evoked spikes in L5B attributed to resting potentials closer to threshold than in L4, so the adult phenotype may not require further experience-dependent alteration.

That experience-driven LTP increases the divergence of TC axons to such a high level would seem to preclude a role in fine tuning feature encoding in L4 cells by strengthening connections only from those TC cells which display the same feature preferences.

The model presented here also provides a simple explanation for the role of sensory experience in axon arborization. If transient synapses are being formed on new and also transient axon branches, LTP may stabilize not only the synapse but also the axon branch on which it is formed. Thus, experience leads to a functional increase in TC divergence with a structural increase in axon arborization and this is supported by the correlated physiological and anatomical findings of decreased TC connectivity in our sensory deprivation experiments. Interestingly, it has been recently reported that whisker trimming results in reduced TC axon branching in adult rodents (Wimmer et al. 2010; Oberlaender et al. 2012). In addition, this may represent an alternative mechanism of axonal plasticity, which would explain the substantially smaller reduction compared with our findings in juvenile mice. Additionally, cortex-specific deletion of GluN1 results in an alteration in TC axon complexity, suggesting a similar link between postsynaptic plasticity and presynaptic axon branching (Lee et al. 2005).

In conclusion, we have demonstrated that sensory experience drives the selective strengthening of the TC to L4 over L6, via a novel mechanism in which plasticity results in a specific increase in divergence of TC connectivity, rather than strengthening individual connections. This provides the mechanism that establishes critical features of the TC input to the barrel cortex and may also account for similar features of this input observed in other primary sensory areas of neocortex.

## Supplementary Material

Supplementary material can be found at: http://www.cercor.oxfordjournals.org/.

## Funding

This work was supported by Medical Research Council Grant MRC (grant G0900461/1) to M.D. Funding to pay the Open Access publication charges for this article was provided by an RCUK block grant to the University of Edinburgh.

## Supplementary Material

Supplementary Data
