# Supplementary material for: Experience-Dependent, Layer-Specific Development of Divergent Thalamocortical Connectivity
Source: Cereb Cortex. 2014 Mar 7;25(8):2255–66. doi: 10.1093/cercor/bhu031 (PMC4494033; doi:10.1093/cercor/bhu031)
Supplement: Supplementary Data [file supp_25_8_2255__index.html]

Experience-Dependent, Layer-Specific Development of Divergent Thalamocortical Connectivity — Experience-Dependent, Layer-Specific Development of Divergent Thalamocortical Connectivity — Supplementary Data 

# Experience-Dependent, Layer-Specific Development of Divergent Thalamocortical Connectivity

## Supplementary Data

Supplementary Data

**Files in this Data Supplement:**

- Supplementary Data - Doc file
- Supplementary Figure 1 - EPS file
- Supplementary Figure 2 - EPS file
