# Supplementary material for: Experience-Dependent, Layer-Specific Development of Divergent Thalamocortical Connectivity
Source: Cereb Cortex. 2014 Mar 7;25(8):2255–66. doi: 10.1093/cercor/bhu031 (PMC4494033; doi:10.1093/cercor/bhu031)
Supplement: Supplementary Data [file supp_bhu031_bhu031supp.doc]

**Figure S1** **TC input to L5B is weak relative to L4 during first postnatal week**

**A**. Example traces showing average of 15 TC EPSCs (upper) and EPSPs (lower) in simultaneously recorded cells from mice aged P3-5 in L4 (black) and L5B (grey). **B.** As for A but from mice aged P7-9. **C**. Summary graph showing change in EPSC amplitude throughout first week in L4 (black) and L5B (grey). **D**. Summary graph showing change in EPSP amplitude throughout first week. * indicates P < 0.5, ** indicates p < 0.01, *** indicates p < 0.005.

**Figure S2 LTP can be expressed at corticocortical synapses on L6 cells.**

**A.** Summary graph of all LTP experiments in L6 cells showing rise time vs % potentiation. Grey points represent fast average values for fast, TC and slow, CC synapses. **B**. Summary graph showing corticocortical (slow) EPSC amplitude in L6 cells in all LTP experiments.
